# Practitioner's Bookshelf

**Published:** 1891-11-21

**Authors:** 


### PRACTITIONERS' BOOKSHELF.

### ARCANA FAIRFAXIANA.

From all students of old medical remedies and past housewifery love at first sight will be won by this russet-boarded volume, with its fair breadth of page and its manuscript text in legible black letter. The subject-matter set forth consists chiefly of quaint recipes which were tried and approved by various members of the Northumbrian Fairfax family during the seventeenth and eighteenth centuries. These receipts became comprised in a written manual of domestic lore, handed down as a precious heirloom through several generations of the said family until it finally reached a lumber chest belonging to Messrs. Gilpin, chemists, of Newcastle-on-Tyne, where it has lain *perdu* until lately, for nearly a hundred years. The venerable tome in its time-worn leather binding was rescued from the dust bin of the Gilpins seven years ago when alterations of their premises were being made; and its contents were found to possess a charm and value sufficient to warrant its reproduction in the present attractive form.

* *Arcana Fairfaxiana.*—A Manuscript Volume of Apothecaries' Lore and Housewifery, nearly three centuries old; used, and partly written by the Fairfax family, of Bilbrough, Yorkshire, during the time of Henry the VIII. With an Introduction by George Weddell. (Newcastle-on-Tyne: Mason, Swan, and Morgan. 1890.)

To begin with, it gives some account of the Fairfax (fair-haired) family, and their genealogy from the time of the first Lord Fairfax, the famous Parliamentary General in the reign of Charles the First. One Mary Cholmeley, whose emblazoned initials are impressed on the cover of this fascinating book, was married in 1626 to the Honourable and Rev. Henry Fairfax, rector of Ashton, and son of the first Lord. She appears to have set great store by the cherished volume, and to have carried it with her to her new home. Afterwards her clerical husband, who ministered much to the sick, made large additions to it in his own characteristic handwriting. And Mary also entered within its pages her private collection of receipts for baking meats, bleaching yarn, and other homely arts. The body of the manual contains a number of shrewd curative formulæ, and practical housewifery suggestions, most of which must have been patiently tested, and found fully worthy before securing the distinction of being thus enrolled. Others are the mere flotsam and jetsam of the convoy, betraying superstition and ignorance, now amusing enough, concerning matters of every day health and hygiene. To wit, we find for "bleeding at ye nose," a method of cure marked "probatum," and which must therefore have been held formerly in high esteem; this was to hang about the "neck in a sattene bagg a toade died in Marche; and by God's grace the flux would stanch presently." Again, "for the emrods (or pyles) it is ordered to burn into powder the hoofe of a horse foote, mixing the same with redd scarlet and white frankincense, then cast the powder on a chafing dish of coles, and sit over them." For the "flos unguentorum—the flower of all oyntments—which shall cure all manner of maladies, aches, or hurts, new or olde—virgin wax and olibanum are to be boyled in white wine; and while this is yet blood warme, the compounder should be for ever stirring, and should then put thereto a quarter of a pound of turpentine. When the mass is cold enough he shall make it up in roles, and keep it to use as the most pretious salve that can be invented. The same 'hath come of Jesus Christ to a Recluse by an Angell, at Red Hill, in Almaine.'" Who can tell but that this primitive recipe gave afterwards a golden hint of superlative value to the Messrs. Holloway for the composition of their famous panacea, said to be manufactured for the most part of beeswax and turpentine? Among receipts of a more domestic sort we notice the Marquis of Granby's instructions how to brew small beer, and Betty Hopper's renowned method of making "mince pyes." Queen Elizabeth's remedy for the wind—poor woman! which served to comfort her royal stomach—consisted of cinnament, ginger, nutmeg, and cloves: such as are reputed to endow Simon the Cellarer with a jolly red nose in the popular song of our present times.

Certain other prescriptions of a more trustworthy and sensible character are to be gleaned moreover from the text of this singular volume. For instance, a sure remedy for toothache, if it do proceed from heat, is taught by squeezing two or three drops of juice from fresh plantain leaves into the party's "contrary eare, and before you can tell to twenty the cure is done!" And here the injunctions of recent medicine are manifestly forestalled. These complacently direct as a late discovery that for inflammatory earache and facial neuralgia the succus of Plantago major (the greater plantain) may be dropped once or twice into the ear, with immediate relief to the patient. And many another reliable hint of equal worth can be culled from the entertaining publication before us, though, to be sure, on the flyleaf is inscribed an apologetic distich of quaint purport, to save the reader from any serious disappointment:—

"Si vis curari de morbo nescio quali,

Accipias herbam, sed quale nescio, nec quâ

Ponas, nescio quo—curabere, nescio quando!"

"Your sore—I know not what—be not fore slow
To cure with herbs, which, when, I doe not know;
Place them (well pounc't) I know not where, and then,
You shall be perfect whole—I know not when."

